# Cultural Orientation and Its Associations with Alcohol Use by University Students in China

**DOI:** 10.1371/journal.pone.0165858

**Published:** 2016-11-02

**Authors:** Shiyuan Wang, Ian M. Newman, Duane F. Shell

**Affiliations:** Department of Educational Psychology, University of Nebraska-Lincoln, Lincoln, Nebraska, United States of America; Harvard Medical School, UNITED STATES

## Abstract

Cultural orientation is defined as an individual’s cultural preferences when encountering imported culture while still living in the native culture. Data was analyzed from 1305 Chinese university students attending universities in Beijing, Kunming, and Wuhan. Cultural orientation was assessed with the Chinese Cultural Orientation Questionnaire, which assesses both Western and Traditional Chinese cultural orientations. The analysis used hierarchical logistic regression with nondrinkers as the reference group and controlling for demographic factors (age, gender, and urban/rural background). Western cultural orientation was found to significantly increase the odds of recent drinking. The results indicated that higher Western cultural orientation was, after gender, the second most important factor associated with Chinese college student drinking frequency. Traditional Chinese cultural orientation was not associated with drinking frequency. This study highlights an unexpected outcome of globalization on students who have not left their home cultures.

## Introduction

Traditionally in China, drinking alcohol is both a normal part of the daily diet, especially in rural areas, and an important part of rituals, business occasions, festivals, and special events. Moderate drinking on important occasions is encouraged, but excessive drinking is discouraged through various social sanctions. The ability to consume alcohol without visible effects is admired, but visible intoxication is considered a personal shortcoming and a disgrace for one’s family and colleagues [1–3]. The one exception to this generalization may be business drinking, where drinking challenges and intoxication are part of the business negotiation [2,4]. The implicit rules associated with alcohol use in much of Chinese society, while different from Western rules, may actually encourage lower-risk behaviors. Rehm et al. [5] expressed concern that globalization is potentially leading to the breakdown of these lower-risk indigenous drinking patterns as individuals who are exposed to influences of Western cultures may be introduced to risky alcohol behaviors, such as binge drinking, bar drinking, and viewing intoxication as fun.

Cultural orientation is an individual’s identification with traditional cultural values and imported foreign cultural values. Cultural orientation reflects an individual’s cultural preferences as he or she encounters influences imported from an outside culture, such as Western values, while still living in and surrounded by his or her native culture. Foreign influences typically come from mainstream and social media, travel, contact with foreigners, use of foreign products, and trying foreign foods, fashions, and celebrations. As a result, a person’s core cultural values (orientation) are challenged and may change. Cultural orientation differs from acculturation, which occurs when an individual emigrates to another country and adopts aspects of the new country’s culture. Today, globalization makes changes in cultural orientation more likely, especially in countries like China that are experiencing new and increasing exposure to foreign influences. Some researchers [6] suggest it be called “globalization-based acculturation”, but we prefer the term orientation because it builds on the earlier work of Eide and Acuda [7] who first coined the phrase “cultural orientation.” Because cultural orientation measures individual orientation towards both the indigenous and foreign cultures, it is useful for examining how cultural influences impact drinking behaviors in the presence of conflicting cultures. This paper explores the associations of cultural orientation with alcohol use by Chinese university students.

Cultural orientation and its contribution in explaining some of the variances in individual alcohol use by young people was first reported the 1990s by Eide and Acuda [7–9]. Eide et al.[8] defined cultural orientation in terms of behavioral indicators, such as an interest in Western, nontraditional practices. Their study of the relationship of cultural orientation to alcohol use among high-school students in Zimbabwe described Western cultural orientation as being associated with a higher probability of alcohol use, while a Zimbabwean orientation was associated with a lower probability of alcohol use [7]. Cultural orientation has been largely overlooked in studies of the effects of globalization on alcohol use in China. However, studies in China by Xue et al.[10], Qian et al.[11], Shell et al.[12], and Tang et al.[13] found that Chinese high-school and college students who expressed higher Western cultural orientation were more likely to consume alcohol. This study sought to extend this prior work by examining, in a different sample of students, the associations of cultural orientation with college student drinking in China while controlling for known drinking influences.

### The Present Study

The current study examined the associations of cultural orientation with alcohol use among Chinese university students. The principal hypothesis for this study was that a more Western cultural orientation would be associated with more frequent drinking. Our objective was not to propose cultural orientation as the most important factor in explaining drinking patterns, but to provide a different perspective on the role of cultural influences in drinking behavior. Many variables influence drinking behaviors. To examine the association of cultural orientation with drinking *beyond* prominent known influences, we controlled for age [14], gender[15] and rural-urban hometown[16].

The results of this study are both empirically and practically significant in an era of increasing globalization. Empirically, they expand our understanding of the associations between cultural influences and drinking behavior among university-aged students. Previous studies of cultural orientation in China have primarily focused on high school students[10–12], and have shown that students with a more Western cultural orientation had a higher likelihood of drinking compared to younger students with a more Chinese cultural orientation. The only previous study of cultural orientation and drinking behavior among college students had similar results to the high-school student studies, but it was limited to only one city [13]. The present study involved surveying students from several universities in three geographically distinct areas of the country. The replication of Tang’s findings [13] in a different and more geographically diverse sample would provide additional empirical evidence of the effects of cultural orientation on drinking behavior and strengthen the external validity of these results. This area of research is important to further understand drinking behavior by young people in an era of globalization by increasing awareness of cultural orientation as a potentially significant factor to be considered in developing alcohol and public health policies in the world’s most populous country and by contributing to the knowledge being assembled to direct efforts to reduce alcohol’s overall contribution to the burden of disease.

## Method

### Ethical Considerations

The ethical review for research with human subjects was conducted by the IRB of the University of Nebraska-Lincoln (Approval #20110411707EP). The UNLIRB waived informed consent procedures. Officials from the participating universities in China approved the study.

### Sampling and Data Gathering

A total of 1,439 students completed the questionnaire. Students were enrolled in 13 universities in Beijing (northern China), Kunming (southwestern China), and Wuhan (central China) and were from hometowns all over China. Trained data collectors gathered the data in existing undergraduate classes. All students in attendance on the survey day completed the survey. The survey, written in Chinese, included a cover page informing the students of the purpose of the study, instructions on how to complete the scales, an assurance of anonymity and confidentiality, a statement of their right to not answer questions without affecting their standing, and a notification that the university authorities had approved the project. Approximately equal numbers of students in each university completed the survey.

Prior to analysis, questionnaires with missing responses on more than half of the cultural orientation items or with contradictory answers in the alcohol use questions were eliminated. The total remaining questionnaires 1,305 (91%) were included in the analysis. There was no specific pattern of demographics in the 134 surveys dropped from the analysis that would affect the result of the analysis. The final sample was 40% male and 60% female, ages between 17 and 30 years (M = 20.24, SD = 1.73). Of these, 62% were freshmen, 15% sophomores, 11% juniors, 6% seniors, and 6% graduate students. Sixty-four percent of the students were from urban hometowns (cities and counties), and 35% were from rural areas (towns and villages).

### Cultural Orientation Questionnaire

The instrument used in this study was a revised version of the Chinese Cultural Orientation Questionnaire (CCOQ) developed by Shell et al.[12] and Xue [10,17,18]. The CCOQ was originally developed for use with high-school-age students. Based on interviews with university students Wang[19] created the current university student version, which was then used by Tang et al.[13]. Details of scale development and complete psychometric and validation information for the CCOQ are available from these sources.

The final cultural orientation questionnaire consisted of 67 items that were answered on a 5-point Likert scale: 1 = strongly disagree; 2 = disagree; 3 = neither disagree nor agree; 4 = agree; 5 = strongly agree. The Chinese cultural orientation factor was measured with five sub-factors: *respect for elders* (“I would listen until elders finish talking even though I disagree”), *filial piety* (“I would do whatever my parent wants me to do”), *pride in being Chinese* (“I am proud of China’s long history”), *collectivism* (“I give priority to group interests rather than individual ones”), and *harmony* (“I won’t show off my strengths to avoid upsetting others”). The Western cultural orientation factor was measured with four sub-factors: *dating attitudes* (“I feel uncomfortable seeing couples show intimacy in public”), *interest in Western culture* (“I appreciate the context of Western culture that emphasizes freedom and taking it easy in life”), *consumerism* (“I would rather buy something that makes me happy than deposit money in the bank”), and *personal appearance preference* (“I like to dye my hair like Westerners’ hair”). A confirmatory factor analysis with these nine first-order factors and two second-order factors was used. Based on Hu & Bentler’s[20] two-index presentation strategy (SRMR and a supplementing index such as RMSEA), two fit indices RMSEA = .047< .06, and SRMR = .065< .08 indicated a good model fit. (In addition, χ2 (1530) = 5886.244, *p*< .05 and CFI = .673 did not show good fit; however, χ2 is sensitive to sample size and model complexity. The SRMR with one other fit index was adequate for model evaluation based on the two-index presentation strategy). In addition, the correlation between the two cultural orientations was nonsignificant, confirming the bidimensional model theory that assumes an individual’s orientations toward the two cultures are independent. Therefore, the two second-order cultural orientations were used as two separate continuous variables in the following analysis (as in Shell et al. [12]). Factor scores for the two second-order orientations (Chinese and Western) were then generated automatically from the model by Mplus. The Cronbach’s α for Chinese cultural orientation was .83, and for Western cultural orientation, it was .80.

### Drinking Measures

Drinking frequency was obtained by asking students to report their drinking in both the last 30 days and the last 12 months. Students were divided into three drinking categories: nondrinkers (had not used alcohol in the 12 months), drinkers (had drunk in the last year but not in the last 30 days), and recent drinkers (had drunk alcohol in the last 30 days). This classification is broad, resulting in only three drinking categories, but our experience in China has been that narrower definitions become increasingly inaccurate for a number of reasons. In China, there is no standardized alcohol serving size, no standardized alcohol container size, greater variability in the percentage of alcohol by volume in different drinks, and drinking practices involving frequent topping up, mixing different drinks on a drinking occasion, and encouraging others to drink, making it difficult to recall what was actually consumed [21,22]. Drinking alcohol is legal for Chinese university students. There is a law prohibiting sale of alcohol to people under age 18, but it is largely ignored. There is no minimum age law for possessing or consuming alcohol.

Demographic questions asked students for their gender, age, and hometown (to identify students from rural and urban backgrounds).

## Results

### Demographic Characteristics and Cultural Orientation

A one-way ANOVA (Table 1) showed that males and students from urban areas had significantly lower Chinese cultural orientation (Mean = -.03, *F* = 8.22 for male; Mean = -.01, *F* = 5.93 for urban) and higher Western cultural orientation (Mean = .02, *F* = 4.87 for male; Mean = .04, *F* = 40.30 for urban).

**Table 1:** Cultural orientation differences by hometown and gender.

	Chinese Cultural Orientation				Western Cultural Orientation			
	Mean (*SD*)	MS	*df*	*F*	Mean (*SD*)	MS	*df*	*F*
Hometown								
Urban	-.01 (.28)	.44	1	5.93*	.04 (.30)	3.48	1	40.30**
Rural	.03 (.26)				-.07 (.28)			
Gender								
Male	-.03 (.30)	.62	1	8.22*	.02 (.31)	.43	1	4.87*
Female	.02 (.26)				-.02 (.29)			
Drinking category								
Nondrinker	-.01 (.29)	.05	2	.50	-.06 (.30)	1.73	2	20.07**
drinker	.01 (.24)				-.03 (.28)			
Recent drinker	-.01 (30)				.07 (.30)			

Note

* significant at .05

** at .01.

For the drinking behaviors, 19% of the sample had not consumed alcohol in the 12 months (nondrinkers), 42% had consumed alcohol in the last 12 months but not in the last 30 days (drinkers), and 39% had consumed alcohol in the last 30 days (recent drinkers). The three types of drinkers showed significant differences in Western cultural orientation, with the recent drinkers group having the most Western orientation (*M* = .07, *F* = 20.07), but no significant difference in their Chinese cultural orientation.

### Associations of Cultural Orientation with Alcohol Use

For the main research question, we used a hierarchical logistic regression [23] to assess the association of cultural orientation with alcohol use *above* other common factors known to be associated with drinking. With nondrinkers as the reference group (Table 2), a block of demographic indicators (age, gender, and urban/rural hometown) were entered into the model in step 1, and then the two cultural orientation factors (Chinese and Western) as a block were entered in step 2, in which the three demographic variables became the controlling block.

**Table 2:** Hierarchical logistic regression.

Drinking Category			β	S.E	Wald	p.	OR	95% C.I for OR	
Step 1	Drinker vs. nondrinker	Intercept	-.68	1.04	.43	.51			
		Age	.07	.05	1.76	.19	1.07	[.97, 1.18]	
		Gender (f = 0, m = 1)	.28	.18	2.61	.11	1.33	[.94, 1.88]	
		Home (urban = 0, rural = 1)	.07	.16	.16	.69	1.07	[.78, 1.47]	
	Recent drinker vs. nondrinker	Intercept	-2.87	1.06	7.30	.01			
		Age	.13	.05	6.41	.01**	1.14	[1.03, 1.26]
		Gender (f = 0, m = 1)	1.36	.18	58.23	< .01**	3.88	[2.74, 5.49]	
		Home (urban = 0, rural = 1)	.47	.18	7.17	.01**	1.60	[1.13, 2.25]	
Step 2	Drinker vs. nondrinker	Intercept	-.65	1.04	.39	.53			
		Age	.07	.05	1.74	.19	1.07	[.97, 1.18]	
		Gender (f = 0, m = 1)	.29	.18	2.73	.10	1.34	[.95, 1.89]	
		Home (urban = 0, rural = 1)	.04	.17	.06	.80	1.04	[.75, 1.44]	
		Chinese CO	.23	.29	.62	.43	1.26	[.71, 2.21]	
		Western CO	.32	.27	1.36	.24	1.37	[.81, 2.33]	
	Recent drinker vs. nondrinker	Intercept	-2.68	1.07	6.26	.01			
		Age	.13	.05	5.89	.02*	1.14	[1.03, 1.26]	
		Gender (f = 0, m = 1)	1.34	.18	55.15	< .01**	3.80	[2.67, 5.41]	
		Home (urban = 0, rural = 1)	.33	.18	3.40	.07	1.39	[.98, 1.98]	
		Chinese CO	.10	.31	.10	.75	1.10	[.61, 2.01]	
		Western CO	1.31	.29	20.52	< .01**	3.71	[2.10, 6.54]	

Note: OR: odds ratio. Step 1 χ^2^ = 114.58**, Step 2 χ^2^ = 143.13**

*significant at .05 level

** significant at .01 level.

When comparing a student’s odds of being a drinker with the odds of being a nondrinker, none of these variables was significant in either step 1 or step 2. However, as seen in step 1, when comparing the odds of recent drinking to nondrinking, age (OR = 1.14, *p* = .01), gender/being male (OR = 3.88, *p*< .01), and urban hometown (OR = 1.60, *p* = .01) all significantly increased the odds of recent drinking. In step 2, after the two cultural orientation variables were added, age (OR = 1.14, *p* = .02) and gender/being male (OR = 3.80, *p* < .01) were still significant predictors of drinking behaviors. Therefore, compared with the odds of nondrinking, participants’ odds of recent drinking increased by 1.14 times with each increase in age of one year; the odds of male students being recent drinkers were 3.80 times those of female students. Western cultural orientation (OR = 3.71, *p*< .01) also significantly increased the odds of recent drinking, by 3.71 times for every point scored higher on the Western cultural orientation scale. By contrast, Chinese cultural orientation did not significantly associate with the odds of recent drinking (OR = 1.10, *p* = 0.75). Hometown became non-significant (OR = 1.39, *p* = .07) after adding the two cultural orientation variables in step 2.

## Discussion

This study suggested that cultural orientation explains additional variance in young Chinese people’s drinking behaviors beyond the expected variances explained by age, gender, and urban/rural residence. This study extends our understanding of cultural influence on drinking behaviors. Previous studies have suggested that, at the country level, as China became more westernized, the traditional Chinese patterns of drinking of its population would erode [2, 24]. This study showed such a trend at the individual level, measured as the association of cultural orientation and drinking in Chinese university students. The results showed that even taking age, gender, and urban/rural background into account, Western cultural orientation was the second most important factor, after gender, associated with Chinese college students’ drinking. The more a student adopted a Western cultural orientation, the more likely he/she was to be a recent drinker. Our results extend the findings of Xue et al.[10, 18] and Shell et al.[12] among Chinese high-school students to university students, and add empirical support for the findings of Tang et al.[13] with a more diverse sample. The sample is not representative of all Chinese university students, but obtaining such a sample for an initial study, like the one presented here, is not practical. In addition, our results are similar to those from earlier studies from Zimbabwe[7–9] about the positive relationship between Western cultural orientation and drinking. In this sense, our study provided additional empirical evidence for the need to consider cultural influences on alcohol use, especially in regions of the world that have drinking patterns and drinking traditions that differ from what is found in North America and Europe.

The socio-demographic characteristics of this sample were consistent with samples in previous studies of alcohol use by this age group in China [15,16]. The male university students in our sample drank more than the females did. Simultaneously, the male students also had higher scores on Western cultural orientation than did the female students. This could suggest that there are both direct and indirect associations of gender on drinking through cultural orientation, which is consistent with Shell et al.’s findings [12]. Drinking increased with age among this group of students. The positive association of age with drinking was different from that in some of the previous literature [14, 25], which made inferences for the entire adult population. Such an inconsistency may suggest that the relationship between age and drinking is not linear. It is possible that drinking increases with age among young people and decreases when people get older. The urban/rural difference in drinking behavior (step 1) disappeared after adding cultural orientation (step 2). However, urban students also had lower scores on Chinese cultural orientation and higher scores on Western cultural orientation compared to rural students. This suggests that differences in rural/urban drinking could be the result of rural/urban differences in cultural orientation, assuming that rural populations are more insulated from foreign influences than are urban populations. This suggests a more complicated picture of the socio-demographic characteristics and drinking behaviors when considering the possible role of culture in drinking.

So far, all the studies on the influence of cultural orientation on drinking behaviors in China, including the current study, have looked at drinking frequency. It is possible that cultural orientation could also be associated with the quantity and type of alcohol consumed. In our study, the socio-demographic characteristics and cultural orientation scores failed to differentiate between drinkers and nondrinkers, while Chinese cultural orientation showed no association with recent drinking. It is possible that there is an association between Chinese cultural orientation and the quantity of people’s drinking; however, as stated earlier, accurate quantity measures are very difficult to obtain. There are no agreed-upon standard drink serving size or standard alcohol containers, there is considerable variability in alcohol by volume, and Chinese drinking practices, such as topping up and mixing different types of alcohol on a single drinking occasion, all make it difficult to report with accuracy the quantity of alcohol consumed. This limitation weakens our conclusion about cultural orientation and drinking behaviors, suggesting that future studies need to develop questionnaires to quantify alcohol intake. In addition, future studies may wish to explore the associations between cultural orientation and different types of alcohol, by including a question on beverage preference: beer, wine or spirits.

### Implications

Rehm et al.[5] expressed concern that globalization is leading to the emergence of less favorable drinking patterns, especially in some regions such as the Western Pacific Region that includes China which have country-level drinking patterns that are less detrimental than those in other regions of the world [26]. This study, at the individual level, suggests a role that cultural orientation may play in the changing patterns of university students’ drinking. Cultural orientation provides an added perspective to the general understanding of cultural influences and acculturation because it reflects cultural values that are present in individuals who have not left their home environment but appear to have been influenced by “outside” cultural forces. Where indigenous cultural values are associated with lower risk patterns of drinking, alcohol policies and education programs may wish to consider how to reinforce the cultural values that reduce alcohol-related risks—values such as moderation, drinking with meals, and viewing intoxication as social embarrassment. Education programs can also consider challenging higher risk drinking patterns of foreign cultures, especially when these other cultures are seen as appealing.

## Supporting Information

S1 Dataset Cultural Orientation and Drinking Data University Students China 2016 Wang. (XLSX)

## References

[1] Hanson DJ. *Preventing alcohol abuse*: *alcohol*, *culture and control* Westport, CT: Praeger Publishers; 1995.

[2] Hao W, Chen H, Su Z. China: alcohol today. Addiction. 2005;100:737–741. 10.1111/j.1360-0443.2005.01036.x 15918802

[3] Hao W, Young D. Drinking patterns and problems in China. J Subst Use. 2000;5:71–78.

[4] Yuan J-M, Ross R, Gao Y-T, Henderson B, Yu M. Follow up study of moderate alcohol intake and mortality among middle aged men in Shanghai, China. Br Med J. 1997;314:18–23. 9001474 10.1136/bmj.314.7073.18 PMC2125578

[5] Rehm J, Rehn N, Room R, Monteiro M, Gmel G, Jernigan D, et al The global distribution of average volume of alcohol consumption and patterns of drinking. Eur Addict Res. 2008;9:147–156. 10.1159/000072221 12970583

[6] Chen SX, Benet-Martinez V, Harris Bond M. Bicultural identity, bilingualism, and psychological adjustment in multicultural societies: immigration-based and globalization-based acculturation. J Pers. 2008;76:803–838. 10.1111/j.1467-6494.2008.00505.x 18482355

[7] Eide AH, Acuda SW. Cultural orientation and adolescents’ alcohol use in Zimbabwe. Addiction. 1996;91(6):807–814. 8696244

[8] Eide AH, Acuda SW, Khan N. Aaroe LE, Loeb ME. Combining cultural, social and personality trait variables as predictors for drug use among adolescents in Zimbabwe. J Adolesc. 1997;20:511–524. 10.1006/jado.1997.0106 9368129

[9] Eide AH, Acuda SW, Roysamb E. Cultural orientation and alcohol-type preferences among adolescents in four sociocultural subgroups in Zimbabwe. J Cross Cult Psychol. 1998;29(2):343–357. 10.1177/0022022198292005

[10] Xue J.P., Newman I.M., Shell D.F., and Fang X.Y. [An analysis of correlative factors of high school students’ alcohol use]. Zhongguo Wiesheng Tongji. 2007;24(4):386–387. Chinese.

[11] Qian L, Hu T, Newman IM, Hou PS. [Study on the relationship between cultural orientation, alcohol expectancy, self-efficacy, and drinking behavior among senior high school students in two cities in Henan province]. Zhonghua Liuxingbingxue Zazhi. 2008;29(3):235–239. Chinese. 18788520

[12] Shell DF, Newman IM, Fang X. The influence of cultural orientation, alcohol expectancies and self-efficacy on adolescent drinking behavior in Beijing. Addiction. 2010; 105:1608–1615. 10.1111/j.1360-0443.2010.03006.x 20626375

[13] Tang H, Cai W, Wang H, Zhang Q, Qian L, Shell DF, et al The association between cultural orientation and drinking behaviors among university students in Wuhan, China. PLoS ONE. 2013;8(1):e54796 10.1371/journal.pone.0054796 23359611 PMC3554628

[14] Pan S, Fang C, Malaga J. Alcoholic beverage consumption in China: a censored demand system approach. Appl Econ Lett. 2006;13:975–978.

[15] Xing Y, Ji C, Zhang L. Relationship of binge drinking and other health-compromising behaviors among urban adolescents in China. J Adolesc Health. 2006;39:495–500. 10.1016/j.jadohealth.2006.03.014 16982383

[16] Ma Y, Zhang B, Wang H, Du W, Su C, Zhai F. [The trend of alcohol use in nine provinces in China from 1993 to 2006]. Zhonghua Yu Fang Yi Xue Za Zhi. 2011;45:323–329. Chinese. 21624327

[17] Xue J. Western cultural influence on alcohol use among adolescents in China [masters thesis]. Lincoln: University of Nebraska-Lincoln; 2002.

[18] Xue J. Cultural orientation and Chinese adolescents' drinking practices [dissertation]. (Order No. 3208121, The University of Nebraska—Lincoln). ProQuest Dissertations and Theses: 2006.

[19] Wang S. Cultural Orientation and Drinking Behaviors among Chinese University Students [masters thesis]. Lincoln: University of Nebraska-Lincoln; 2011.

[20] Hu L, Bentler PM. Cutoff criteria for fit indexes in covariance structure analysis: conventional criteria versus new alternatives. Struct Equ Modeling. 1999;6(1):1–55.

[21] Newman IM, Qian L, Xue JP. [College student alcohol surveys: The need for uniform questions]. Zhongguo Yaowu Lanyong Fangzhi Zazhi. 2004;10(5):272–275. Chinese.

[22] Newman IM, Qian L, Zhang JG, Zhao J, Zhang Y. [Review of studies of Chinese high school students’ drinking behavior]. Zhongguo xue xiao wei *sheng* 2009;30(12):1139–1143. Chinese.

[23] Pedhazur EJ. Multiple Regression in Behavioral Research (3rd ed.). FL, Orlando: Harcourt Brace; 1997.

[24] Colon I. Homeland, gender and Chinese drinking. J Addict Disease. 1997;13(2):59–67. 10.1300/j069v13n02_05 8204675

[25] Kim JH, Lee S, Chow J, Lau J, Tsang A, Choi J, et al Prevalence and the factors associated with binge drinking, alcohol abuse, and alcohol dependence: a population-based study of Chinese adults in Hong Kong. Alcohol Alcohol. 2008;43:360–370. 10.1093/alcalc/agm181 18230698

[26] World Health Organization. Global status report on alcohol and health 2014 Geneva, Switzerland: WHO; 2014 Retrieved from http://www.who.int/substance_abuse/publications/global_alcohol_report/en/

